# The gut microbial composition in polycystic ovary syndrome with insulin resistance: findings from a normal‐weight population

**DOI:** 10.1186/s13048-021-00799-9

**Published:** 2021-03-27

**Authors:** Fangfang He, Yumei Li

**Affiliations:** Department of Assisted Reproduction, Xiangya Hospital, Central South University, 410008 Changsha, People’s Republic of China

**Keywords:** Polycystic ovary syndrome, Gut microbiota, Insulin resistance, *Enterococcus*

## Abstract

**Background:**

Limited studies have reported the relationship between intestinal flora dysbiosis and clinical characteristics in polycystic ovary syndrome **(**PCOS). However, the structure and characteristics of gut microbiota in PCOS have not been fully elucidated.

**Objective:**

To analyze the composition of the Intestinal flora population in normal-weight women with PCOS and insulin resistance(IR) compared to PCOS alone and healthy women.

**Methods:**

A total of 14 PCOS patients with insulin resistant(PCOS-IR) and 12 PCOS alone (PCOS-NIR), and 10 age- and body mass index-matched healthy control women (HC). BMI: 18.5–23.9 kg/m^2^. The bacterial 16 S rDNA V3-V4 fragment was amplified and sequenced. Then, the sequencing data were analyzed for species annotation, community diversity, and inter-group differences, to explore gut microbial characteristics of the subjects and their correlation with clinical parameters.

**Results:**

No significant difference in diversity was observed between PCOA and sample cluster analysis among the three groups (Beta-diversity) and Alpha-diversity. The relative abundance of *Rothia, Ruminococcus, and Enterococcus* was significantly higher in the PCOS-IR group than in the other two groups (P < 0.05), while that of *Prevotella* was dramatically decreased (*P* < 0.05). The abundance of *Enterococcus* was positively correlated with waist circumference, hip circumference, diastolic blood pressure, and insulin resistance index. Meanwhile, *Rothia* abundance is positively associated with waist circumference and free fatty acids.

**Conclusions:**

The gut microbial composition of PCOS patients with insulin resistance is different from that of PCOS alone and healthy women. The difference is correlated with the clinical characteristics of PCOS, with regards to insulin resistance, abdominal obesity, free fatty acids, and other indicators. PCOS-IR patients have an increased abundance of *Enterococcus* which potentially the intestinal environment of the host by enriching the metabolic pathways related to insulin resistance, causing the occurrence and development of PCOS.

## Introduction

Polycystic ovary syndrome (PCOS) is a common endocrine and metabolic disease in women, related to hirsutism, hyperandrogenism, ovulation dysfunction, menstrual disorders, and infertility [1]. About 50-70 % of cases of anovulatory infertility in patients are linked to PCOS [2], specifically those accompanied by low ovulation induction rate, low pregnancy rate, and high abortion rate. PCOS is associated with several metabolic disorders, including insulin resistance (IR), obesity, cardiovascular disease, diabetes, and other long-term metabolic syndromes. At present, the etiology and pathogenesis of PCOS are still unknown, which may involve lifestyle, neuroendocrine, genetic factors, immune and metabolic dysfunction [3]. Insulin resistance is considered to be the main pathological basis of reproductive dysfunction in polycystic ovary syndrome,independent of obesity [4–8]. Notably, insulin resistance and hyperinsulinemia affect testosterone synthesis and secretion, while hyper androgen levels can lead to hirsutism, acne, ovulation disorders, and menstrual disorders. On the other hand, insulin resistance exhibits a long-term and severe effect on metabolism in patients with polycystic ovary syndrome [9].

Intestinal flora,the “second genome” obtained by the human body, co-evolves with the host to promote metabolism and immune response [10]. Evidence indicates that gut microbiome disorders are closely related to the occurrence and development of metabolic diseases, including PCOS [11, 12]. Also, several studies have attributed the appearance of IR to gut microbiota dysbiosis [13, 14]. The changes of intestinal flora potentially affect insulin sensitivity by regulating chronic inflammation mediated by lipopolysaccharide, branched chain amino acid, short chain fatty acid (SCFA), and bile acid metabolism, and stimulating the secretion of enterocerebral peptides, resulting in insulin resistance and hyperinsulinemia [15–17]. The structure and characteristics of intestinal microbiota in patients with PCOS have not been comprehensively elucidated [18–22]. Besides, only limited studies have explored the relationship between intestinal flora dysbiosis, clinical characteristics and metabolism in patients with PCOS.

Intestinal flora imbalance causes IR, which is closely linked to the occurrence of PCOS. Herein, excluding the influence of obesity, we conducted a pilot study to examine the correlation between gut microbiota, insulin resistance, and clinical characteristics of PCOS patients. These findings might provide novel insights on the mechanism of occurrence and development of PCOS, thus, accelerating the formulation of new approaches for the prevention and treatment of PCOS.

## Materials and methods

### Participants

A total of 26 women with polycystic ovary syndrome (PCOS) aged 18–35 years who visited the Department of Assisted Reproduction (Xiangya Hospital, Central South University) between August and December 2019 were recruited. Additionally, we recruited 10 normal women visiting a similar department during the same period for assisted reproduction due to the “male-factor” or “fallopian tube factor” were enrolled as the control group. This study was approved by the Ethics Committee of the Department of Assisted Reproduction (Xiangya Hospital,Central South University)and China Registered Clinical Trial Ethics Review Committee (Ethical Review No.: CHiECRT1900028223). All participants provided written informed consent. The participants were non-obese women (BMI 18.5–23.9 kg/m^2^) [23, 24]. PCOS diagnosis was conducted as per the Rotterdam criteria revised during the 2003 Conference; PCOS was diagnosed if two out of the following three features were present: Oligoovulation or amenorrhea; clinical manifestations of androgen and/or biochemical hyperandrogenism (HA); polycystic ovary: ultrasound reports show that the follicles of unilateral or bilateral ovaries with a diameter of 2-9mm are larger than 12, and/or the volume of the ovary ≥ 10ml [7]. Individuals excluded from one of the following conditions: Cushing syndrome, congenital adrenocortical hyperplasia, androgen-secreting tumors. None of the subjects were treated with hormone drugs, insulin sensitizers, antibiotics, probiotics and prebiotics, traditional Chinese medicines, and immunosuppressants less than three months before the study. Based on the Rotterdam criteria, women in the control group had no history of menstrual disorder, endocrine diseases, or diagnosed PCOS. The HOMA-IR index was calculated as follows: fasting insulin (FINs, mIU/L)๡fasting plasma glucose (FPGs, mmol/L)/22.5. Insulin resistance was defined as fasting insulin > 10mU/ml, or HOMA-IR > 1.66, or abnormal insulin release curve (insulin peak more than ten times the basic value; insulin peak delayed to 1 h after taking sugared water; area under the insulin curve increased; insulin level not returning to the normal fasting level 3 h after taking sugared water), but with normal fasting blood glucose and glucose tolerance levels [25].

### Sampling

Data regarding anthropometry and metabolic parameters were collected for all participants, including: (1) measurements, including height, weight, waist circumference, hip circumference, and blood pressure, (2) detection of biochemical indicators, such as the sex hormone levels, including progesterone (P_4_), prolactin (PRL), testosterone(T), luteinizing hormone (LH), follicle-stimulating hormone (FSH), oestradiol (E2), sex hormone binding globulin (SHBG), Anti-Mullerian hormone (AMH), and glucose and insulin levels during an oral glucose tolerance test on the third day of the menstrual cycle (In the case of amenorrhea patients, blood samples were collected on any day of the menstrual cycle). Meanwhile, biochemical indices, including triglyceride (TG), total cholesterol (TC), low-density lipoprotein (LDL), high-density lipoprotein (HDL), total bile acid, free fatty acid, and inflammatory markers such as C-reactive protein (CRP), interleukin-6 (IL-6), and tumor necrosis factor (TNF-α) were measured. (3) Feces samples were collected from all patients after menstruation, and the samples were immediately frozen and stored at -80℃ until analysis.

### DNA extraction and PCR amplification

Following the manufacturer’s instructions, microbial DNA was extracted from stool samples using a TIANgen stool DNA kit. The 16 S rDNA V3 + V4 region of the ribosomal RNA gene was amplified by polymerase chain reaction (PCR). The V3–V4 variable region of the bacterial 16sRNA was amplified via PCR using (5′-CCTACGGRRBGCASCAGKVRVGAAT-3′) and (5′-GGACTACNVGGGTWTCTAATCC-3′) primers under the following conditions: denaturing at 94 °C for 3 min followed by 24 cycles of 94 °C for 5 s, 57 °C for 90 s, and 72 °C for 10 s, then a final extension at 72 °C for 5 min. Each PCR reaction mix (25 µL) included 2.5 µL TransStart Buffer, 2 µL dNTPs, 1 µL primer (2 µM), 0.5 µL TransStart Taq DNA and 20 ng of DNA template. The PCR products were assessed using 1.5 % agarose gel electrophoresis, quantified by Qubit3.0 Fluorometer (Invitrogen, Carlsbad, CA), then pair-end sequenced on Illumina Miseq PE250 platform (Illumina, San Diego, CA, USA).

### Next-generation sequencing

Filtration of raw tags was performed using QIIME (V1.9.1) to dislodge the noisy sequences [26]. The filtered clean tags were searched against the Gold database to identify chimeric tags, which were then removed using the UCHIME algorithm to obtain Effective Tags. Based on the Effective Tags of each sample, the OUT (Operational Taxonomic Units) were clustered using QIIME software (Version 1.9.1) based on the GreenGene database [26–28]. Alpha diversity was reflected by Chao1, Observed OTUs,Simpson, and Shannon indexes. Beta diversity was presented on principal coordinates analysis (PCoA) charts created using PCoA statistical analysis method with R language(version 3.3.3). Based on the species abundance table, the *P*-value was obtained by Kruskal–Wallis H test analysis and then modified by Benjamini and Hochberg False Discovery Rate method to obtain the Q value. The species with significant differences between groups were obtained with Q value < 0.05 as the threshold [29]. Linear discriminant analysis (LDA) effect size (LEfSe) was used to identify the bacterial taxa and metabolism-associated clinical parameters with significant differences between groups. Logarithmic LDA values > 2.0 and *P* < 0.05 were set as the threshold for differential flora identification.

### Statistical analysis of clinical data

Data were analyzed using SPSS 22.0. Quantitative demographic and clinical data with normal distribution were presented as the mean ± standard deviations (SD). Unpaired t-test was used to determine the difference between two groups. Qualitative demographic and clinical data were expressed as percentages and analyzed using the chi-square test. The Kolmogorov-Smirnov test of normality was applied to all data sets. Data with non-conformance to normal distribution were analyzed using the Mann-Whitney test. A probability (p) value of < 0.05 was considered statistically significant.

## Results

### Clinical characteristics of the participants

Table 1 summarizes the clinical, hormonal and metabolic data of the recruited participants. There were no significant differences(*P* > 0.05) in age, height, or BMI among the three groups among the three groups. The menstrual cycle, waist circumference, and waist-to-hip ratio of the PCOS group were significantly higher than those of the healthy control group(HC). The waist-to-hip ratio of the PCOS-IR group was higher than that of the other two groups (IR VS NIR: 0.85 ± 0.04 VS 0.82 ± 0.03, IR VS HC:0.85 ± 0.04 VS 0.77 ± 0.03 *P* < 0.05). Regarding sex hormones, patients with PCOS exhibited higher levels of T, LH, LH/FSH, and lower levels of estradiol, relative to the control patients (*P* < 0.001). Nonetheless, no significant difference was observed the PCOS-IR and the PCOS-NIR group. Concerning glucose and plasma lipid levels, although fasting glucose was not different among the three groups, women with PCOS had higher fasting insulin, glycosylated hemoglobin, and homeostasis model assessment of insulin resistance (HOMA-IR). Notably, the PCOS-IR group had higher HOMA-IR values(*P* < 0.05). In contrast healthy individuals, the plasma levels of pro-inflammatory cytokines in PCOS patients, including IL-6 and TNF-a, were significantly higher. The PCOS-IR group showed higher levels of C-reactive protein, Il-6, TNF-α, and free Fatty acid (FFA)(*P* < 0.05).

**Table 1:** Clinical, biochemical and hormonal features of participants

		PCOS	
**Parametes**	**HC(*n* = 12)**	**NIR(*n* = 10)**	**IR(*n* = 14)**
**Age(years)**	**28.25 ± 1.22**	**26.4 ± 3.41**	**26.71 ± 2.43**
**Menstrual cycle(days)**	**29.67 ± 2.02AB**	**82.7 ± 26.61B**	**75.43 ± 27.91 A**
**BMI(Kg/m2)**	**21.28 ± 1.34**	**21.32 ± 1.22**	**21.91 ± 1.4**
**Waist**	**67.42 ± 4.4AB**	**71.8 ± 3.29BC**	**77.79 ± 5.73AC**
**Hip**	**88 ± 5.31**	**87.8 ± 3.33**	**91.54 ± 4.43**
**Waist to hip ratio**	**0.77 ± 0.03AB**	**0.82 ± 0.03BC**	**0.85 ± 0.04AC**
**FPG (mmol/L)**	**5 ± 0.27 A**	**5.09 ± 0.47**	**5.42 ± 0.59 A**
**FINs(uU/mL)**	**6.91 ± 1.82 A**	**6.95 ± 2.52 C**	**14.81 ± 3.88AC**
**HOMA-IR**	**1.54 ± 0.45 A**	**1.61 ± 0.64 C**	**3.57 ± 1.07AC**
**HbA1-C**	**5.04 ± 0AB**	**5.37 ± 0.21B**	**5.26 ± 0.28 A**
**FSH (IU/L)**	**6.8 ± 2.18**	**5.92 ± 2.16**	**6.43 ± 1.42**
**LH(IU/L)**	**6.25 ± 2.88AB**	**15.55 ± 8.03B**	**15.37 ± 7.31 A**
**LH/FSH**	**0.96 ± 0.4AB**	**2.53 ± 0.89B**	**2.42 ± 1.2 A**
**T (nmol/L)**	**0.75 ± 0.21AB**	**1.93 ± 0.57B**	**1.81 ± 0.82 A**
**P(nmol/l)**	**0.54 ± 0.16B**	**0.93 ± 0.52B**	**0.74 ± 0.43**
**E2(pmol/L)**	**183.51 ± 100.9B**	**281.47 ± 164.67B**	**194.25 ± 55.9**
**AMH**	**4.99 ± 3.74 A**	**7.99 ± 2.79**	**9.28 ± 5.12 A**
**TBA (umol/l)**	**4.7 ± 2.22**	**5.51 ± 4.77**	**5.26 ± 2.4**
**ALT (U/L)**	**13.18 ± 2.7**	**21.02 ± 15.13**	**20.81 ± 11.77**
**AST(U/L)**	**18.22 ± 5.91**	**22.03 ± 6.72**	**21.64 ± 6.4**
**Lithic (umol/L)**	**243.43 ± 37.04B**	**320.21 ± 56.66B**	**283.65 ± 105.62**
**TG( mmol/L)**	**0.88 ± 0.25 A**	**1.14 ± 0.84**	**1.29 ± 0.41 A**
**TC( mmol/L)**	**4.29 ± 1.11**	**4.39 ± 0.68**	**4.77 ± 0.88**
**HDL(mmol/L)**	**1.47 ± 0.31**	**1.36 ± 0.26**	**1.33 ± 0.23**
**LDL(mmol/L)**	**2.67 ± 0.59**	**2.6 ± 0.53**	**2.9 ± 0.6**
**APO-a**	**93.13 ± 71.68**	**120.14 ± 126.97**	**194.49 ± 220.43**
**ApoA/ApoB**	**2.47 ± 0.48**	**2.31 ± 0.56**	**2.22 ± 0.62**
**FFA(mmol/L)**	**0.47 ± 0.11 A**	**0.48 ± 0.31**	**0.66 ± 0.24 A**
**hsCRP(mg/L)**	**0.69 ± 0.56 A**	**0.8 ± 0.75 C**	**1.63 ± 1.27AC**
**IL-6(pg/ml)**	**1.13 ± 0.46AB**	**2.12 ± 0.26B**	**2.56 ± 1.28 A**
**TNF**	**3.76 ± 1.43AB**	**4.86 ± 1.07B**	**4.54 ± 0.51 A**

A: *P* < 0.05 for HC vs. IR-PCOS group

B: *P* < 0.05 for HC vs. NIR-PCOS group

C: *P* < 0.05 for IR vs. NIR-PCOS group

Abbreviation: *PCOS* polycystic ovary syndrome; *PCOS-IR* PCOS with insulin resistance; *PCOS-NIR* PCOS withoutinsulin resistance; *BMI* body mass index; *WHR* the ratio of waist to hip; *FSH* follicular stimulating hormone; *LH*luteinizing hormone; *TNF-a* tumor necrosis factor-a; *FPG* fasting plasma glucose; *FINS* fasting plasma insulin;*HOMAIR* homeostasis model assessment of insulin resistance; *PPG* 3h postprandial plasma glucose; *TC* totalcholesterol; *HDL-C* high density lipoprotein-cholesterol; *LDL-C* low density lipoprotein-cholesterol; *ALT* alanine aminotransferase; *AST* aspartate aminotransferase

### Effect of PCOS on gut microbiota diversity

**Fig. 1:**
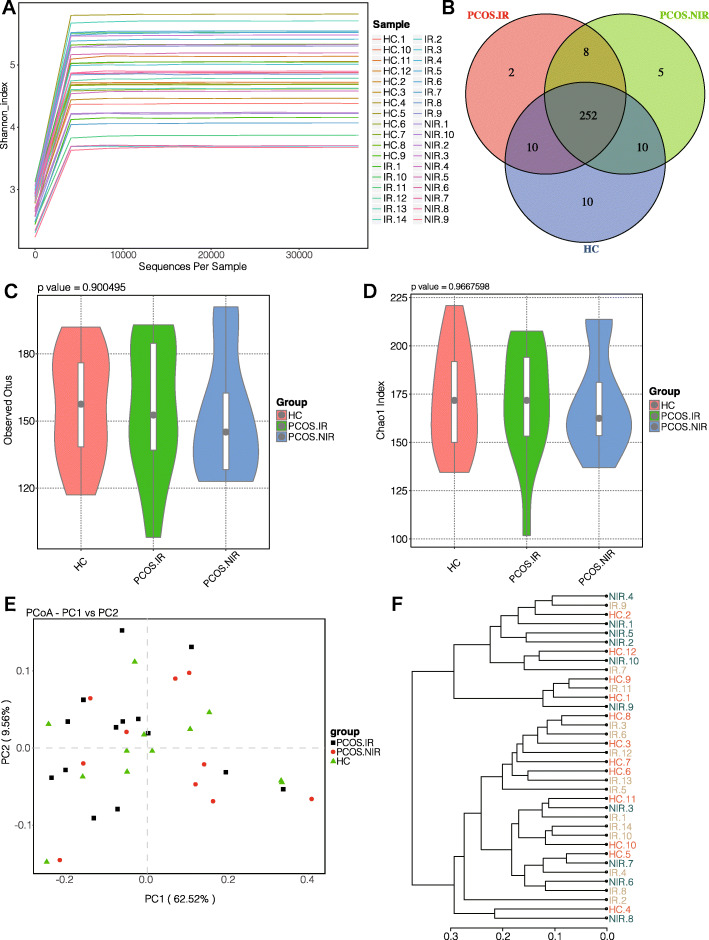
Alpha and beta diversity of the gut microbial communities from participants (*n* = 36). **a**: Shannon–Wiener curves, showing that the amount of sequencing data is large enough to reflect the vast majority of microbial information in the samples. **b**: Venn diagram, displaying the number of common and unique OTUs, and the similarity and overlap of OTUs among groups. **c**: Comparison of observed OUT Numbers between the three groups(*P* > 0.05). **d**: Comparisons of Chao1 indexes among the three groups(*P* > 0.05). **e**: Principal coordinate analysis (PCoA) of fecal microbiota based on weight UniFrac metric, each dot represents the bacterial community composition of one individual stool sample, and the axis titles indicates the percentage variation explained (62.52 and 9.56 % respectively). **f**: Dendrogram showing hierarchical cluster analysis based on weight UniFrac distance matrix to measure the closeness between individual samples

These sequences comprised 297 OTUs clustered at a 97 % similarity level. The dilution curve and Shannon-Wiener curve were used to establish whether the sequencing quantity was sufficient and estimate the species richness (Fig. 1 a). The curve seemingly flattened, indicating that the sequencing depth was sufficient to reflect the species diversity of the samples. The Venn diagram of OTU number distribution of the three groups of samples was drawn to intuitively reflect the common and unique characteristics between the groups (Fig. 1b). Alpha diversity analysis showed that, compared with the HC group, observed OTUs and Chao1 index were decreased in the PCOS-IR group and the PCOS-NIR group, but the differences were not statistically significant (Fig. 1 c and d). The Beta diversity analysis showed that the principal coordinate analysis (PCoA analysis) and sample clustering analysis were similar among the three groups of samples (Fig. 1e,  f).

### Gut microbiota composition among PCOS patients

**Fig. 2:**
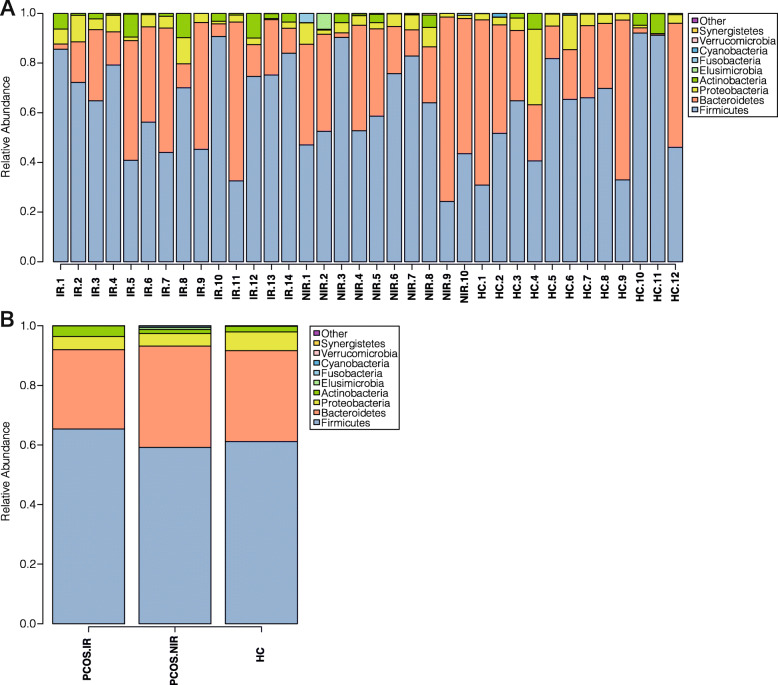
Species analysis of phylum-level differences. **a**: thermal maps of phylum-level flora of each sample (**b**) : Thermal maps of three groups of phylum-level flora

**Fig. 3:**
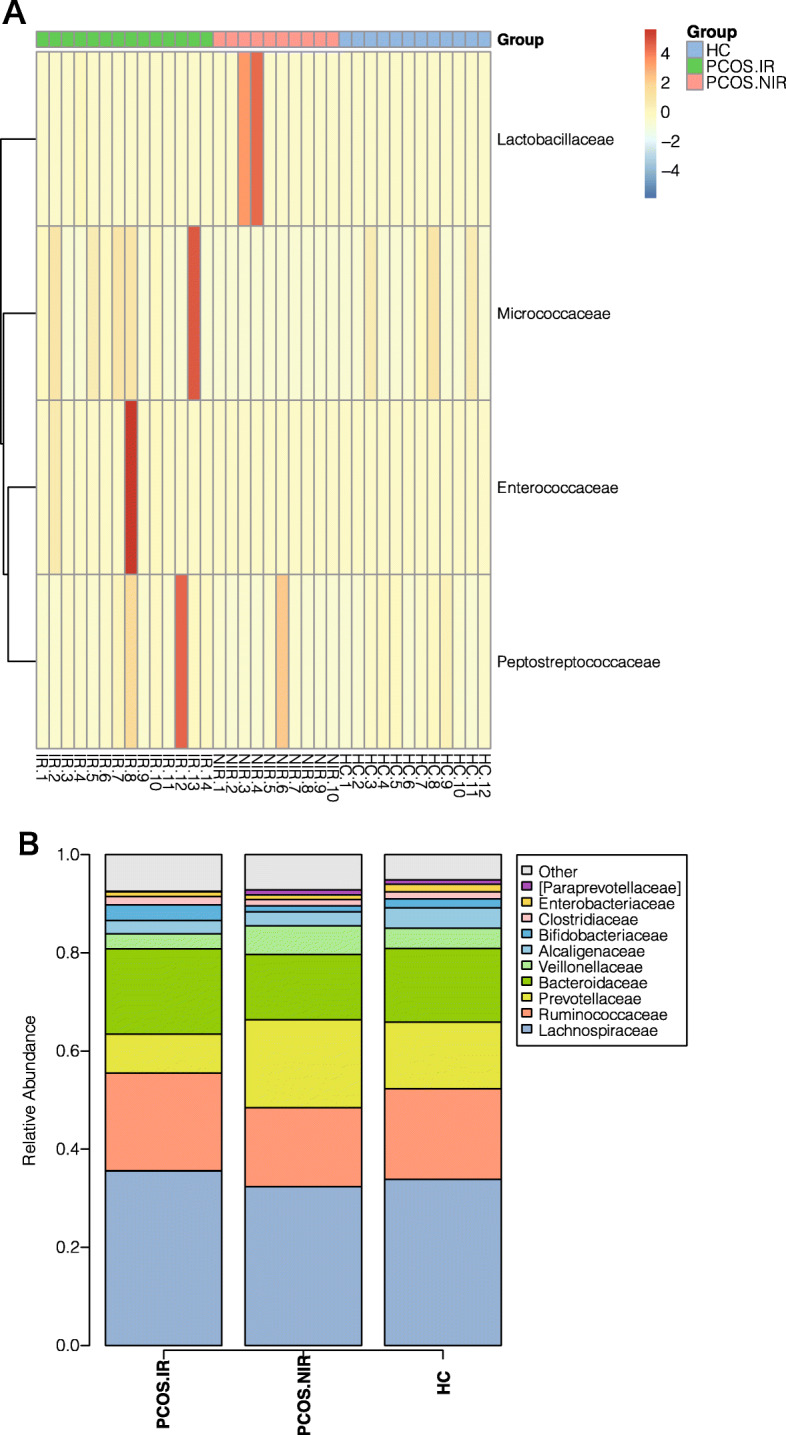
Analysis of different bacterial flora at the family level (**a**): Bar chart of bacterial colony composition between family-level groups (**b**) Thermal chart of bacterial colony abundance at family level differences

**Fig. 4:**
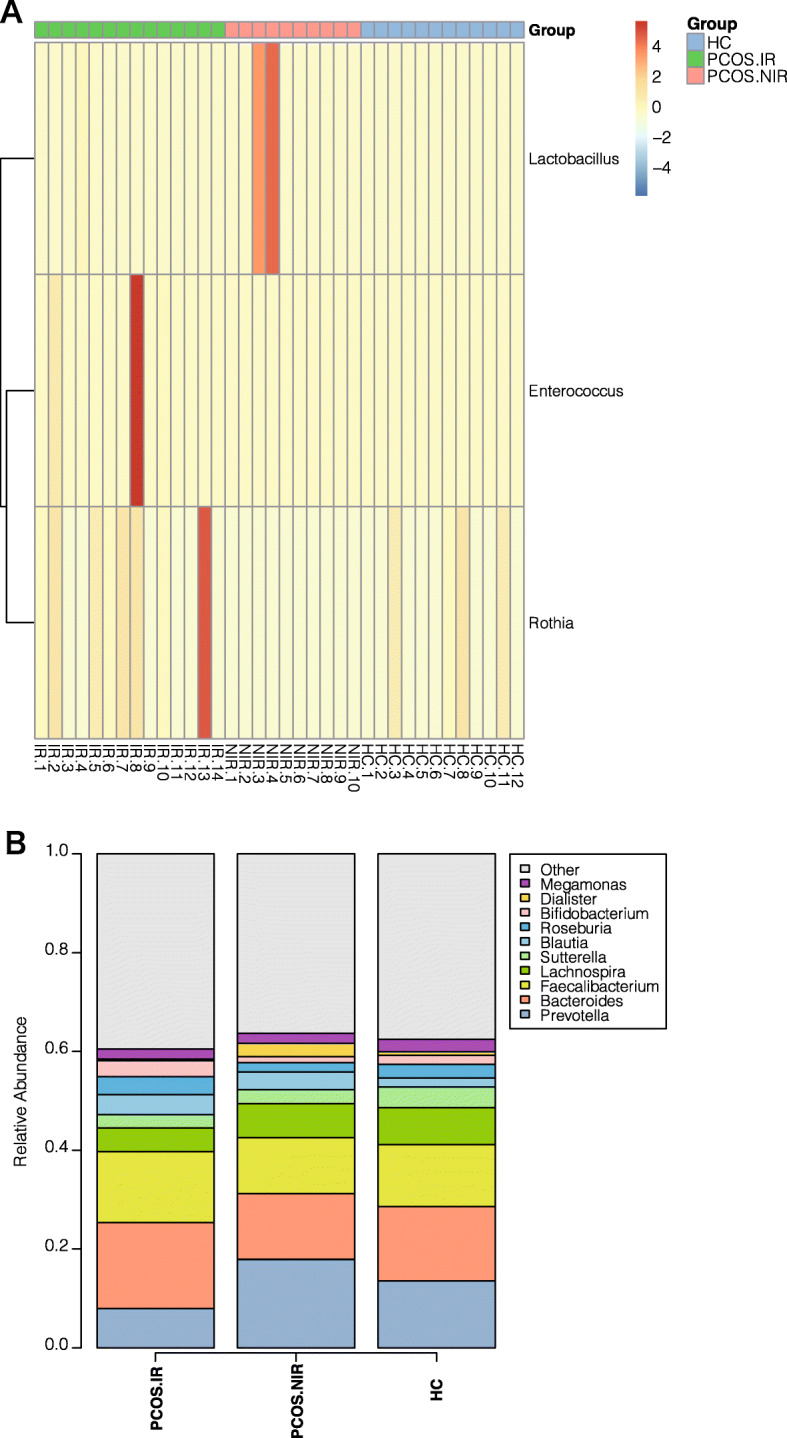
Analysis of bacterial flora with difference in genera level (**a**): Bar chart of bacterial colony composition between genera level groups (**b**): Thermal chart of bacterial colony abundance with difference in genera level groups

At the phylum level, Fig. 2 shows the phylum abundance distribution in the bacterial kingdom of the 36 samples, among which *Firmicutes* were the most abundant, followed by *Proteobacteria*, then *Actinobacteria*. The overall abundance of other bacteria was about 1 %. The abundance of *Fusobacteria* and *Verrucomicrobia* differed between the PCOS and the HC groups, but the difference was not significant (*P* > 0.05) (Fig. 2). At the family level, significant differences were observed in the abundance of *Lactobacillus*, *Enterococcaceae*, *Peptostreptococcaceae*, and *Micrococcaeae* among the three groups. In the PCOS-IR group, the abundance of *Peptostreptococcaceae*, *Enterococcaceae*, and *Micrococcaeae* was higher than that of the other two groups. *Lactobacillaceae* was the highest in the PCOS-NIR group (Fig. 3). Compared with the HC group, PCOS patients exhibited a higher abundance of *Enterococcus*. The relative abundance of *Rothia*, *Ruminococcus*, *Lachnospira*, and *Enterococcus* was significantly higher in the PCOS-IR patients than in the other two groups (*P* < 0.05), whereas the abundance of the *Prevotella* was dramatically decreased (*P* < 0.05). *Lactobacillus* and *Akkermansia* were more abundant in the PCOS-NIR group than in the PCOS-IR and HC groups (*P* < 0.05) (Fig. 4).

**Fig. 5:**
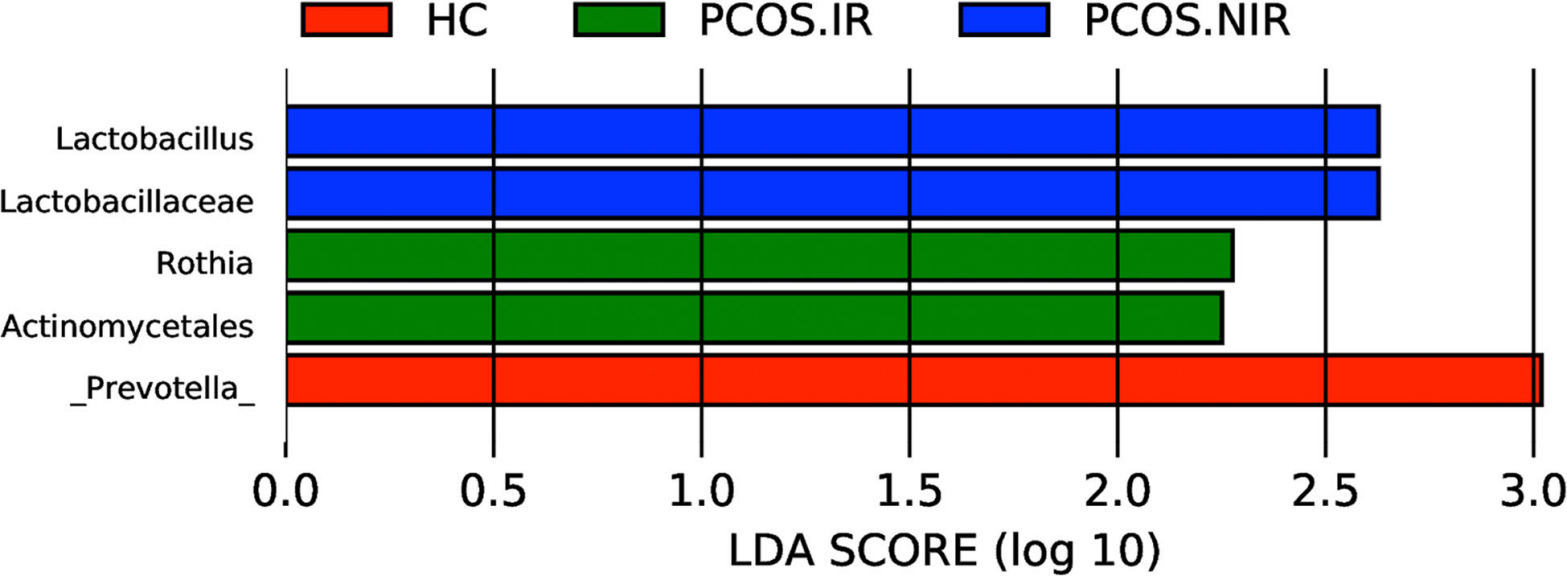
Identification of the bacterial taxa with statistically significant difference between groups using LEfSe software and LDA. Taxa enriched in HC, NIR-PCOS, and IR-PCOS group are colored by red, green, and blue respectively (LDA > 2.0 and *P* < 0.05), the relative abundance of these biomarkers are shown in the histogram (mean and standard deviation values are plotted) under the corresponding cladogram

Next, to further identify species bacterial taxa with significant differences among the three groups, we used LEfSe multilevel species discrimination and LDA. Rothia and Lactobacillus were identified as the characteristic microbiota in the PCOS-IR and the PCOS-NIR groups, respectively, while the dominant bacteria in the HC group was Prevotella(LDA score > 2.0 and *P* < 0.05)(Fig. 5).

### Correlations between gut microbiota and metabolic parameters or sex hormones

**Fig. 6:**
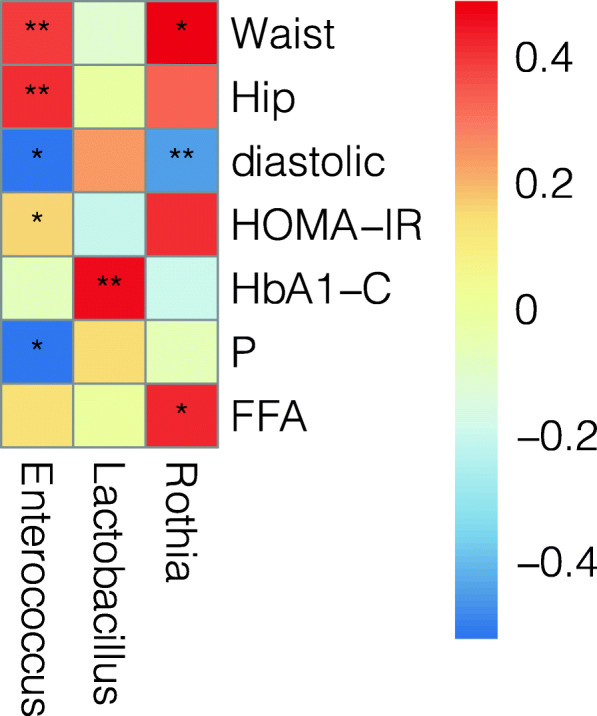
Spearman correlation heat map between clinical parameters and flora. The color of spots represents the Spearman correlation R-value between each bacterial taxa and clinical parameters. + and + + are respectively 0.01 < *P* < 0.05, 0.001 < *P* < 0.01

At the genera level, *Enterococcus* was positively correlated with waist circumference, hip circumference, diastolic blood pressure, and HOMA-IR index. Rothia positively correlated with waist circumference and free fatty acid (FFA) (*P* < 0.05) (Fig. 6).

## Discussion

Gut microbial plays a vital role in regulating energy storage and human metabolism. As such, substantial focus has been directed to microbiota-targeted agents as novel targets for the treatment of polycystic ovary syndrome (PCOS) and related metabolic diseases. Several studies have found variations in gut microbiota composition between PCOS patients and healthy people. Besides, obese PCOS patients have been shown to exhibit more severe gut dysbiosis. However, the precise mechanism underlying the relationship between gut microbiota and the occurrence and development of PCOS remains significantly unreported. Insulin resistance (IR) is critical pathological basis of reproductive dysfunction in patients with PCOS [4–7]. Exactly 50 % of patients with PCOS have IR, independent of obesity [30, 31]. However, there is limited information on the roles played by intestinal flora in the development of IR and its link with PCOS. Herein, our results revealed that gut dysbiosis was more severe in PCOS patients with insulin resistance than in the PCOS-NIR and HC groups. Furthermore, several taxa at the phylum level were related to the clinical characteristics of PCOS and were significantly correlated with metabolic biomarkers, including HOMA-IR, abdominal obesity, free fatty acids, and other indicators.

Moreover, we showed that the composition of gut microbiota of PCOS patients with normal BMI was changed, but there was no significant difference in α-diversity among the three groups. Noteworthy, several studies demonstrated conflicting results regarding the composition and function of the intestinal flora in PCOS patients. According to a recent meta-analysis, decreased intestinal microbiome diversity and changes in diversity are closely associated with obesity in humans [32, 33]. Previous research reported a significant decrease in gut microbiota diversity in PCOS patients or letrozole-induced mouse models [19, 20, 34]. Liu et al. found that obese PCOS patients had the lowest diversity of gut microbiota [19]. Meanwhile, studies have shown that sex hormones are related to changes in gut microbiom [16, 35]. Torres et al. found that PCOS patients exhibited a lower diversity of gut microbiota than healthy controls, and that total testosterone levels were associated with reduced diversity [20]. However, Insener et al. did not find a decrease in the diversity of gut microbiota in all PCOS patients with hyperandrogenemia, whether obese or not. and diversity may be different from PCOS diagnostic criteria [21]. Similarly, in this study, β- diversity of gut microbiota did not significantly differ among the three groups of samples based on weighted and unweighted clustering analysis. In a recent study, weighted UniFrac range-based hierarchical clustering and PCoA analysis indicated a clear distinction between the HC and the IR groups, whereas the NIR group could not be distinguished from the HC and IR groups [22]. Despite the absence of standard selecting sample size in microbiome studies, a study estimated that a sample size of 10 subjects per unweighted group (total sample size of 30) and 20 subjects per weighted group (total sample size of 60) might provide accurate statistical results for weighted analysis [36]. Besides, the α and β diversity of intestinal flora may be influenced by sex, sex hormones, and obesity. In this study, the BMI of all the subjects was within the normal range, and thus the effect of obesity itself on the gut microbiota composition of the PCOS patients could not be considered. Maybe due to the small sample size, a more definite answer should be given after increasing the sample size in the future.

According to the analysis of the structural composition of gut microbiota conducted in this study, the three groups were mainly composed of *Bacteroidetes* and *Firmicutes* at the phylum level. At the genus level, the relative abundance of *Enterococcus* in the PCOS group increased significantly (P < 0.05) and was highest in the IR group. *Enterococcus* is a common gram-positive bacteria and can be divided into five categories according to phylogenetic similarity. Among them, *Enterococcus faecalis* and *Enterococcus faecium* are the major pathogenic bacteria in humans [37, 38]. Although the causal role of genus *Enterococcus* in the occurrence and development of metabolic diseases has not been fully revealed, previous studies have found that *Enterococcus* is more abundant in the gut microbiota of obese children and adolescents [39], as well as in mice under a high-fat/high-sugar “Western” diet [40]. A recent study found that *Enterococcus* can regulate the level of incretin hormone glucagon-like peptide-1 (GLP-1). GeIE secreted by *E. fasecalis* can degrade GLP-1 (GLP-1), causing abnormal insulin secretion[41]. At the same time, GeIE can degrade intestinal gastric inhibitory peptides (just like leptin), and thereby interfere with the metabolism of the host. Similarly, in our study, Enterococcus was the most abundant genera in the PCOS-IR group, and its abundance was positively correlated with insulin resistance index. GLP-1 plays a role in regulating glucose homeostasis and reducing appetite in the body. Considering the vital role of GLP-1 in the development of type 2 diabetes and other metabolic diseases, we speculated that *Enterococcus* could influence the occurrence and development of PCOS by regulating the GLP-1 signaling pathway, specifically in patients with IR. Studies have shown that after oral glucose tolerance tests, GLP-1 activity in lean PCOS patients is usually lower than in healthy women [42]. The use of GLP-1 receptor agonists in the treatment of PCOS patients enhances symptoms and reduces metabolic complications by reducing weight and insulin resistance [43, 44]. Insulin secretion and gastric emptying are affected by the intestinal flora environment and intestinal flora imbalance. Estelle et al. proved the important role of intestinal flora in controlling GLP-1-induced insulin secretion and gastric emptying in mice [45]. Therefore, the role of *Enterococcus* in the regulation of GLP-1 level in PCOS patients should be explored further.

The critical bacterial genus in the intestinal tract of patients with PCOS was identified using LEfSe multilevel species discrimination and LDA. Consequently, *Rothia* played vital roles in the PCOS-IR group, whereas *Prevotella* was the dominant bacterial group in the HC group. Rats fed a high-fat diet exhibited significantly increased fat, reduced insulin sensitivity, increased abundance of esophageal *Rothia*, and *Rothia* were associated with fasting blood glucose and insulin. These observations were linked the expression of inflammatory genes and fatty acid transport and metabolism in the esophagus [46]. Changes in *Rothia* flora in the adolescent oral cavity were associated with obesity [47]. Women with gestational diabetes have also shown an increase in *Rothia* abundance, relative to women with normal blood sugar [48]. In this study, *Rothia* had significant advantages in the PCOS-IR group, and its abundance was positively correlated with waist circumference and free fatty acid (FFA). Previous studies have shown that non-obese PCOS patients with insulin resistance have a more remarkable centra distribution of fat [37]. In our research, PCOS patients with normal BMI exhibited abdominal fat accumulation. Besides, *Rothia* abundance in the PCOS-IR group was related to abdominal obesity. Intestinal flora may participate visceral fat metabolism, release excess too much free fatty acids, increase lipotoxicity, and reducing insulin sensitivity. *Prevotella* is a bacterium that produces short-chain fatty acids (SCFA), regulates the uptake of nutrients and hormone levels in the gut. Also, it participates in energy metabolism, and its decreased abundance is significantly associated with increased testosterone and pro-inflammatory cytokines [22]. In this study, the HC group had the highest abundance of *Prevotella*, maintaining the balance of intestinal flora, while the NIR and IR groups had a reduced abundance of *Prevotella*. However, there was no correlation between the decreased abundance of the Prevotella and the sex hormones and biochemical indicators, potentially due to a small sample size used. The relationship between PCOS and intestinal flora is complex and could be related to genetic, lifestyle, and environmental factors [49, 50]. We excluded the influence of smoking, taking antibiotics and probiotics on intestinal flora in the included subjects. However, distinct backgrounds (including region, race, lifestyle, and diet habit) of the subjects could be responsible for the differences in the PCOS-related intestinal flora [51].

## Conclusion

In conclusion, we analyzed the composition of the intestinal microbiota in PCOS patients with normal BMI combined with insulin resistance and its relationship with clinical characteristics. Notably, no significant difference was found in intestinal microbial diversity among the three groups of patients. Nonetheless, the abundance of *Enterococcus* and *Rothia* significantly increased in the PCOS-IR group and correlated with insulin resistance, suggesting that the bacterium potentially plays a key role in the pathogenesis of PCOS by regulating the glucagon-like peptidin-1 (GLP-1) level and other mechanisms. The altered intestinal flora might affect the intestinal environment of the host by enriching different metabolic functions and promoting the development of PCOS insulin resistance and disease in women. This experimental cross-sectional study involved participants from different regions, RACES, and eating habits, which might have brought heterogeneity. Considering the variations in human intestinal microbiota, many clinical population and animal experiments are essential to validate our findings. Also, further studies are imperative to elucidate the precise function of various flora and the mechanism underlying their roles in the pathogenesis of multiple diseases. Therefore our next task will involve performing a metabolomic analysis to explore further the relationship between insulin resistance and intestinal flora among PCOS patients. These findings will provide a theoretical basis for drug design and subsequent clinical treatment of PCOS.

## Data Availability

The datasets are available from the corresponding author on reasonable request.

## Abbreviations

PCOS: Polycystic ovary syndrome
IR: Insulin resistance
BCAA: Branched chain amino acid
SCFA: Short chain fatty acid
HA: Hyperandrogenism
P: Progesterone
PRL: Prolactin
T: Testosterone
LH: Luteinizing hormone
FSH: Follicle-stimulating hormone
E2: Oestradiol
SHBG: Sex binding globulin
AMH: Anti-Mullerian hormone
TG: Triglyceride
TC: Total cholesterol
LDL: Low-density lipoprotein
HDL: High-density lipoprotein
CRP: C-reactive protein
IL-6: Interleukin-6
TNF-α: Tumor necrosis factor
OUT: Operational Taxonomic Units
LDA: Linear discriminant analysis
FFA: Free fatty acid
GLP-1: Glucagon-like peptide-1
